# EGFR-mutant NSCLC presenting with stroke and massive systemic embolization as the first manifestation: case report

**DOI:** 10.1186/s12883-021-02236-2

**Published:** 2021-06-09

**Authors:** Zheng Wang, Jiangyong Miao, Lina Wang, Ying Liu, Hui Ji, Xiangjian Zhang, Lili Cui

**Affiliations:** 1 Department of Respiratory Medicine III, The Second Hospital of Hebei Medical University, Shijiazhuang, Hebei China; 2 Department of Neurology, The Second Hospital of Hebei Medical University, 309 Zhonghuabei Street, Hebei 050000 Shijiazhuang, People’s Republic of China; 3 Hebei Key Laboratory of Vascular Homeostasis and Hebei Collaborative Innovation Center for Cardio-cerebrovascular Disease, Shijiazhuang, Hebei China

**Keywords:** Cancer-related ischemic stroke, Dabigatran, Lung adenocarcinoma, Trousseau’s syndrome, Case report

## Abstract

**Background:**

Presentation with massive systemic embolization as the initial manifestation of occult malignancy is infrequent. The standard management of cancer-related arterial thromboembolism has not yet been established.

**Case presentation:**

We described a case of Trousseau’s syndrome resulting in acute ischemic stroke concomitant with multiple embolizations in the spleen and kidney during oral administration of dabigatran for pulmonary embolism preceding the diagnosis of a malignant tumor. A cancer-related hypercoagulable state was suspected because the patient was admitted to the neurology department due to acute ischemic stroke with three territory infarcts on diffusion-weighted imaging (DWI) in the absence of identifiable conventional risk factors and brain vessel narrowing. The patient was subsequently diagnosed with epidermal growth factor receptor (EGFR) mutation–positive non-small-cell lung cancer (NSCLC) (stage IV) with pleural metastasis. Administration of low-molecular-weight heparin followed by long-term dabigatran under effective cancer therapy comprising gefitinib and subsequent chemotherapy did not cause stroke relapse during the 1-year follow-up.

**Conclusions:**

This case suggests that cancer-related hypercoagulability should be considered an important etiology for stroke patients who develop unexplained disseminated acute cerebral infarction without conventional stroke risk factors, especially concomitant with multiple organ embolization. Novel oral anticoagulants may be an alternative therapy for the long-term management of cancer-related arterial thromboembolism under effective cancer therapy.

## Background

It is well known that patients with malignant tumors have a high risk of developing thromboembolic events [1]. Currently, idiopathic thromboembolic events, including venous and arterial thromboembolism (ATE), that precede the diagnosis of an occult visceral malignancy or appear concomitantly with cancer, are referred to as Trousseau’s syndrome. The risk of venous thromboembolism (VTE) increases approximately 7-fold in patients with malignancy, affecting up to 20 % of the cancer population. Symptomatic arterial thrombosis in cerebral vessels is rare (~ 2-7.4 %) but is still among the most common complications of cancer and is usually one of the most important causes of high mortality [2, 3]. Approximately 10 % of hospitalized ischemic stroke patients have malignant tumors. In 40 % of them, cancer-related coagulopathy contributed to the mechanism of stroke. Approximately 14.6 % of patients with malignancy present pathological evidence of cerebrovascular ischemic events, and approximately 50 % of cancer-associated cerebrovascular diseases are cryptogenic, compared to a 30 % cryptogenic rate in the general population [4]. Concurrently, the prevalence of cancer and stroke appears to increase because of the development of the aging population.

Trousseau’s syndrome presenting with ischemic stroke as the first symptom is infrequent [5]. Cancer-related stroke is often misdiagnosed as embolic strokes of undetermined source (ESUS). Therefore, cancer-associated hypercoagulability (CAH) or embolism is often overlooked by neurologist as a possible cause of ischemic stroke due to the absence of tumor-related symptoms and signs. Even more rarely reported is Trousseau’s syndrome as the presentation of cerebral infarction concomitant with massive systemic infarction as the initial manifestation of occult malignancy [5]. This uncommon presentation and lack of suspicion can lead to a misdiagnosis and delayed treatment with deadly consequences [6]. Patients with CRIS usually receive the same treatment for cerebral infarction as those who do not have cancer, which could worsen their condition and deteriorate their clinical prognosis. The identification of the clinical features of CRIS before the diagnosis of malignancy deserves additional attention from neurologists. Recently, emerging data linking cancer to ischemic stroke have been discussed. Some neurologists even discussed the possibility of CRIS as a new stroke subtype. They suggest that CRIS is as common as established stroke subtypes, such as atherosclerotic and lacunar stroke. Therefore, neurologists should pay much more attention to the etiology of stroke, such as CRIS, especially for patients with ESUS.

## Case presentation

 In November 2019, a 49-year-old female with no smoking history was referred to the cardiovascular division for shortness of breath. Coronary computed tomographic angiography (CTA) showed no atherosclerosis or narrowing of coronary arteries. A computed tomography (CT) scan of the chest revealed pulmonary nodules and a small pleural effusion on the left side. CT pulmonary angiography revealed pulmonary embolism with complete small arterial occlusions. Blood coagulation studies showed extended activated partial thromboplastin time (APTT, 27.4 s; normal: 25.4–38.4 s) and prothrombin time (PT, 13.5 s; normal: 9.4–12.5 s) and significantly increased levels of fibrin degradation products (FDP, 98.35 mg/L; normal: 0–5 mg/L) and D-dimer (2.99 µg/ml; normal: 0 ~ 0.24 µg/ml). Therefore, anticoagulation therapy with oral dabigatran was initiated. However, no further auxiliary examination was performed to identify the potential causes of pulmonary embolism.

**Fig. 1:**
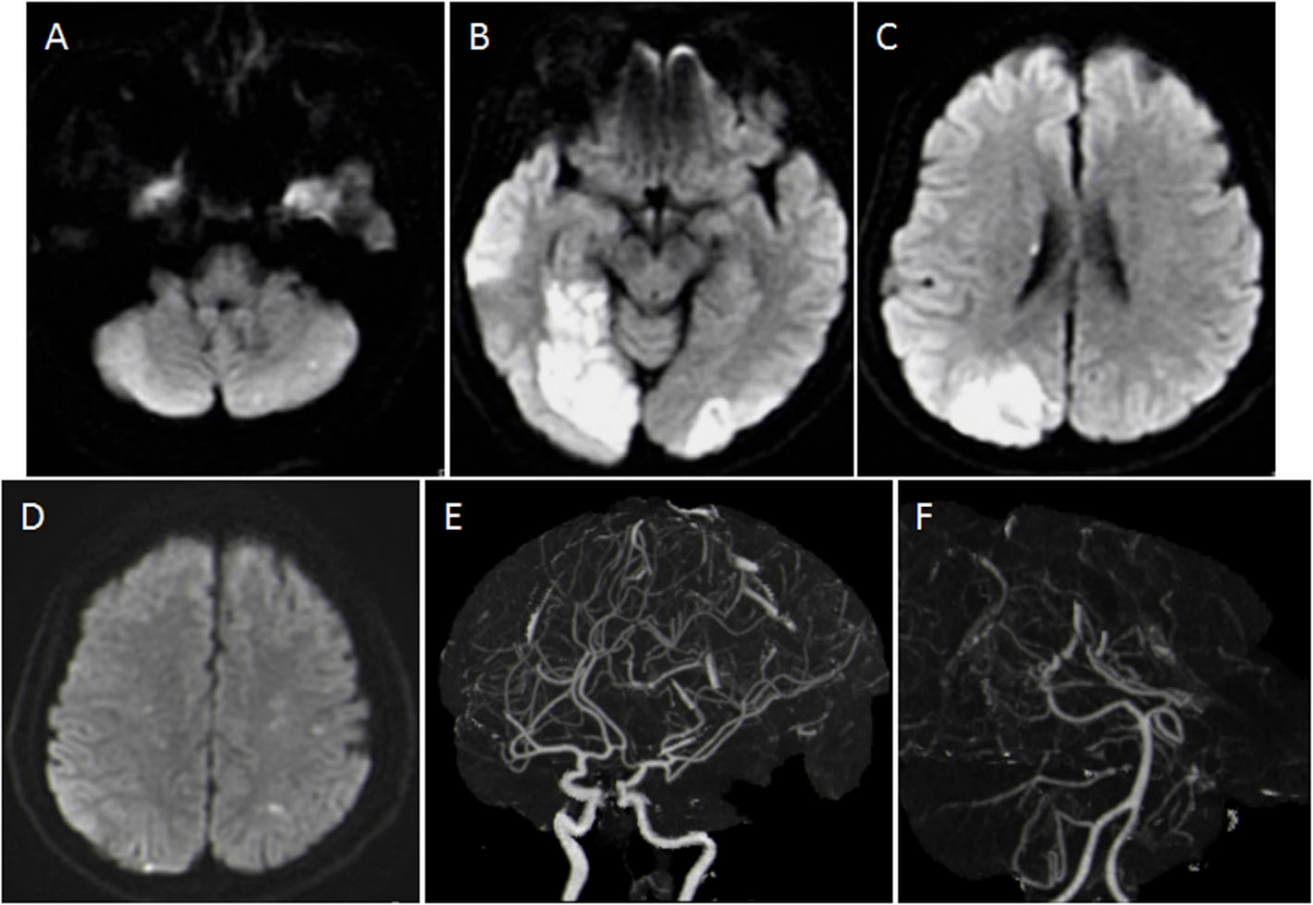
DWI showed multiregional cerebral infarction in “three territories” (**a-d**). Brain CTA images showed no stenosis and occlusion in cerebral arteries (**e** and **f**)

One month later, she was admitted to our neurology department because of non-fluent speech and blurry vision. Neurological examination on admission showed neurologic defects, including motility aphasia and homonymous hemianopsia. No conventional cerebrovascular risk factors were identified. Brain CT scan, magnetic resonance imaging (MRI) and diffusion-weighted imaging (DWI) depicted multiregional acute and subacute cerebral infarcts in bilateral middle cerebral artery territories and bilateral posterior artery territories (Fig. 1). Neither atherosclerotic plaque in the aortic arch nor stenosis or occlusion in major extracranial and intracranial arteries was observed on her upper chest, cervical and brain CTAs. The examination results of carotid duplex ultrasonography were unremarkable. No cardio thrombi or vegetation was seen in transthoracic echocardiography (TTE), and no arrhythmia was detected by Holter monitoring. The bubble study, in conjunction with echocardiogram findings, was indicative of a grade III right-to-left cardiac shunt (RLS), suggesting a risk of paradoxical embolism.

The results of the laboratory examination were almost within the normal range, including serum white blood cell count, red blood cell count, platelet count, hemoglobin, creatinine, cardiac enzymes, alanine aminotransferase and aspartate aminotransferase. The coagulation system showed activation, with an elevated FDP level of 17.7 mg/L, a D-dimer level of 2.59 µg/ml, a PT of 13.5 s, and an APTT of 29.3 s. Because the patient had multiple infarctions involving the bilateral anterior and posterior circulation without obvious risk factors for stroke, we investigated all vasculitic and infection markers. Except for elevated C-reactive protein (CRP, 45.5 mg/L; normal: 0–8 mg/L), the other blood biochemical test results were not abnormal (including immunoglobulins, anti-cardiolipin antibody, antinuclear antibody, anti-SSA antibody, P-ANCA and C-ANCA). The results of subsequent hematologic tests, including protein S, protein C, anti-thrombin III, von Willebrand factor, lupus anticoagulant and anti-β2-glycoprotein antibody, were all within the normal range.

**Fig. 2:**
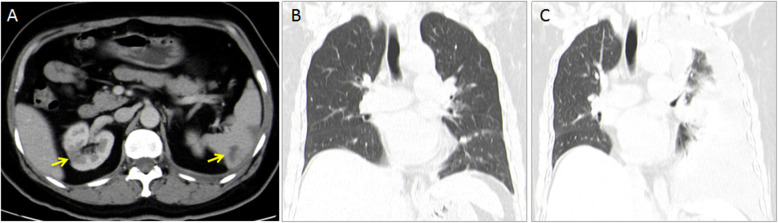
Abdominal CT scan revealed multiple infarction in spleen and kidney (**a**). The CT scan of chest showed increased pleural effusion (**c**) than that of a month ago (**b**)

Multiple cerebral infarctions and pulmonary embolisms made us think of the possibility of CAH status. Interestingly, laboratory studies found high serum levels of tumor markers, including carcinoma antigen (CA) 125 (209.2 U/ml, normal range < 35 U/ml), CA199 (459.3 U/ml, normal range < 37 U/ml), CA 153 (35.95 U/ml, normal range < 25 U/ml), carcinoembryonic antigen (CEA, 48 ng/ml, normal range < 10 U/ml) and neuron-specific enolase (36.8 ng/ml, normal range < 16.3 U/ml). A contrast-enhanced abdominal CT scan revealed infarcts in the spleen and kidney, indicating massive thromboembolic events in multiple visceral organs (Fig. 2). Then, a chest CT scan was performed for the second time and showed increased pleural effusion on the left side compared to one month prior (Fig. 2). Cytological examinations of pleural effusions collected via thoracentesis indicated cancer cells with positive staining of CK7, EMA, Napsin A and TIF-1. As a result of a detailed examination, she was diagnosed with stage IV lung adenocarcinoma expressing L858R-mutant epidermal growth factor receptor (EGFR). 18 F-FDG PET imaging revealed multiple metastatic patterns in intrathoracic metastases, including malignant effusion and simultaneous pleural and pericardial metastases.

Then, an EGFR tyrosine kinase inhibitor (EGFR-TKI), gefitinib, was administered at a dosage of 250 mg/day. Considering the identification of lung cancer, she was diagnosed with Trousseau syndrome with massive infarction in multiple organs. Then, she was treated with low-molecular-weight heparin (LMWH) while in the hospital. After 2 months of treatment with gefitinib, the pleural effusion content was decreased on the thoracic CT scan. Laboratory data showed a decline in the serum levels of FDP (from 17.7 mg/l to 1.01 mg/l) and D-dimer (from 2.59 mg/l to 0.18 mg/l). She transitioned to take dabigatran for secondary prevention of ischemic stroke after discharge because of its convenient purchase and easy oral administration. After treatment with gefitinib for 4 months, the patient showed disease progression with regard to increased pleural effusion. Then, she received systemic chemotherapy comprising intravenous injection of pemetrexed (500 mg/m^2^ body surface area) and cisplatin (75 mg/m^2^ body surface area) at 21-day intervals for up to 4 cycles, followed by monotherapy with pemetrexed for 5 cycles in another oncology hospital. Radiotherapy was not performed on the patient. At the end of follow-up on February 2, 2021, the patient was in good condition with moderate adverse events, including nausea, mild anemia and leukopenia. Laboratory tests on February 2, 2021, revealed a slight decrease in hemoglobin content (90 g/L), normal white blood cells (6,410 per ml), and moderately elevated levels of FDP (4.33 mg/l) and D-dimer (0.278 µg/ml). The CT scan of Chest showed decrease in pleural effusion and indicated the tumor was stable without new metastatic lesions. However, after 6 months of anticoagulant therapy, the patient stopped taking dabigatran for unknown reasons. During the 1-year follow-up, no recurrence of stroke was observed.

## Discussion and conclusions

In this report, we presented a case of multiple cerebral infarctions without significant cerebral vascular stenosis. Cancer-related ischemic stroke (CRIS) was finally considered the etiology of stroke because of her specific clinical features, including the absence of cardiovascular risk factors, multiple infarctions involving different territories, potential hematological biomarkers, and concomitant peripheral organ embolization.

In cancer patients, intravascular coagulation can cause widespread systemic small arterial and venous thrombotic vasculopathy. Although VTE is the most common manifestation, CAH may also present as migratory superficial thrombophlebitis, disseminated intravascular coagulation (DIC), massive ATE, or thrombotic microangiopathy. Population-based evidence demonstrated a cumulative incidence of symptomatic ATE in cancer patients, including myocardial infarction and ischemic stroke, of 4.7 %~ 5.4 % compared with 2.2 % in control patients at 6 months after new diagnoses of cancers [3]. Common sites of cancer-associated ATE include the spleen, kidney, mesentery and extremities, but cerebral and cardiac consequences had the most significant morbidity and were the most likely to reveal the diagnosis.

Cardioembolic manifestation is thought to be an important cause of stroke in cancer patients, with approximately 40 % of CRIS exhibiting a cardioembolic pattern on neuroimaging, compared to 15 %~30 % in the general stroke population. Nonbacterial thrombotic endocarditis (NBTE) was indicated to be the most common cause of embolic stroke in cancer patients in an autopsy study [2]. Adenocarcinoma, including lung, pancreatic, and gastric adenocarcinomas and those of unknown primary sites, is the most frequent histologic type of cancer associated with NBTE. NBTE in malignancy is characterized by the deposition of sterile platelets and fibrin on previously undamaged cardiac valves in the absence of bacterial infection. Vegetation often occurs on the surface of aortic and mitral valves. Right-sided heart valves are less commonly involved [7]. Vegetation varies in size from microscopic to large and is easily dislodged due to the little inflammatory reaction at the site of attachment, therefore increasing the frequency of extensive arterial embolic events and recurrent stroke [8]. It has been reported that nearly 50 % of patients with NBTE have systemic emboli, with cerebral, renal, coronary, and mesenteric circulations being the most frequently involved [7]. A sudden neurological deficit was the most common clinical presentation and often the only evidence of thromboembolism. The pathogenesis of NBTE is not fully determined. DIC and carcinoma mucin promoted coagulation factor activity and the deposition of platelets on cardiac valves, which are the main causes of cancer-related NBTE. Increased circulating inflammatory cytokines and TF in cancer patients as well as autoimmune disease may also result in local valve damage and contribute to the formation of valvular vegetation.

CRIS should be highly suspected in patients with multiregional cerebral infarcts or widely distributed emboli in organs of unknown etiology as well as in those who progress poorly on antiplatelet therapy. High clinical suspicion and the performance of echocardiography to detect the presence of valvular thrombi are required for the diagnosis of NBTE in CRIS. However, the diagnosis of NBTE with TTE is often difficult because the vegetation is usually small and fragile. Transesophageal echocardiography (TEE) is superior to TTE in detecting valvular vegetations as definite or possible sources of ischemic stroke in cancer patients. The yields of TTE and TEE in detecting NBTE were 22 and 62 %, respectively, for suspected cardioembolic strokes [9]. In the present case, although the DWI pattern of multiple infarcts highly suggested a cardioembolic etiology, TTE did not identify any masses attached to mitral or aortic valves. Regrettably, no direct evidence demonstrated the cardiac source of embolization in this patient because we did not routinely perform TEE to detect potential emboli in cancer patients.

The recognition of radiographic patterns of cerebral infarction contributes to a more accurate identification of the etiology and enables the selection of appropriate treatment [6, 10, 11]. In our case, DWI showed multiple small DWI infarcts in the bilateral corona radiate and left cerebellar hemisphere and a large-area infarction in the medial temporal and occipital lobes, indicating massive involvement of the anterior and posterior circulation in an usual pattern. It has been reported that a considerable portion of cancer patients with newly diagnosed cerebral infarction do not have conventional cardiovascular risk factors and more commonly present with multiple infarct lesions (59.8 % ~87 %) on brain imaging [12, 13]. Multiple infarctions shown in different vascular territories on brain DWI are a specific radiologic pattern for CRIS patients. Bilateral cerebral embolisms on DWI imaging were significantly more common in CRIS patients than cardiogenic embolisms and artery-to-artery embolisms [14]. Nouh et al. [15] recently reported the diagnostic value of “Three Territory Sign (TTS)” as a radiographic DWI marker for cerebral ischemic infarct caused by cancer-associated hypercoagulation or NBTE. They reported CRIS as the most frequent etiology of “Three Territory Infarcts (TTIs),” accounting for 20 % of all patients [16]. In the absence of any identifiable embolic source, CRIS accounts for 75 % of cases. TTS is also a more frequent DWI pattern of CRIS than stroke-related atrial fibrillation (AF) for identifying the etiology of ESUS. Acute DWI infarcts in 1- and 2-territory patterns did not show a difference between malignancy and atrial fibrillation etiology. TTS is 6 times more likely to be detected in CRIS than in AF-related infarction [16]. Most TTIs of patients showed multiple small or medium lesions in multiple lobes on DWI imaging [17]. Nevertheless, larger cerebral artery occlusions can also be present because the masses vary in size from microscopic to large (larger than 10 mm), similar to what we have reported in this case [18].

In this case, serum D-dimer and FDP levels showed a trend of persistent increase before the identification of malignancy, accompanied by multiple organ embolism, indicating the hypercoagulable state of this patient. Subsequently, effective anticancer therapy improved the coagulation abnormalities and led to the patient being in good condition during the one-year follow-up. Compared to patients with other types of cerebral infarction, including larger-artery atherosclerosis, cardioembolism, small-artery occlusion and ESUS, patients with CRIS had the highest levels of plasma D-dimer, CRP and FDP [19, 20]. High serum D-dimer levels were associated with early neurological deterioration, stroke relapse and poor prognosis, with a median survival of 4.5 months in CRIS patients [5, 21]. Ito et al. reported a D-dimer cutoff value of 2.0 µg/ml for distinguishing CRIS from conventional stroke etiologies, which had a sensitivity of 71.1 % and a specificity of 82.9 % [19].

The underlying pathophysiology of CAH is characterized by the formation of platelet-rich microthrombi in medium and small vessels. However, the inhibition of endogenous thrombin did not block mucin-induced platelet aggregation. To prevent thrombosis via this mechanism, heparin and LMWH are considered much more effective than vitamin K antagonists such as warfarin because of their ability to prevent both hypercoagulation and platelet aggregation [22]. In this case, due to the inconvenience of purchasing and long-term management, the patient did not continue to use LMWH for secondary prophylaxis of stroke. To date, there are limited data evaluating the efficacy and safety of novel oral anticoagulants in the second prophylaxis and long-term management of recurrent cancer-associated stroke. Current evidence supporting the therapeutic effect of anticoagulation on cancer-related ischemic stroke was obtained from a few small retrospective studies by evaluating reductions in D-dimer levels, recurrent events, and microemboli with treatment [4, 23]. Previously, a few cases of patients with malignant tumors who developed cerebral ATE during novel oral anticoagulant (NOAC) administration for VTE treatment have been reported [24]. These results indicated that NOACs may be insufficient for preventing cancer-related ATE. In our case, the patient was administered NOAC for one month for the treatment of pulmonary embolism before developing cerebral infarction and visceral infarcts. Although NOACs did not prevent ischemic stroke at the initial stage before lung adenocarcinoma diagnosis, no recurrence was observed when the patient continued to use dabigatran during the following course of chemotherapy. This case indicates that NOACs may be effective in preventing cancer-related ATEs under efficient cancer therapy conditions. However, further research is needed to evaluate the efficacy and safety of anticoagulation therapy in the prevention of recurrent ATE in cancer patients.

Patients with CRIS are prone to present widespread systemic ATE and multiregional cerebral infarction in different vascular territories on DWI. The possibility of Trousseau syndrome should be considered an important etiology of ESUS, especially for patients who present with undetermined multiple DWI infarcts concomitant with elevated serum levels of D-dimer and FDP without identifiable conventional stroke risk factors. Our present case revealed that NOACs may be effective and safe in the prevention of cancer-related ATE under effective cancer therapy.

## Data Availability

All data related to this case report are stored in the neurology department of Second Hospital of Hebei Medical University, and are available from the corresponding author on reasonable request.

## Abbreviations

EGFR: Epidermal growth factor receptor
EGFR-TKIs: EGFR tyrosine kinase inhibitors
VTE: Venous thromboembolism
ATE: Arterial thromboembolism
CRIS: Cancer-related ischemic stroke
ESUS: Embolic strokes of undetermined source
CAH: Cancer-associated hypercoagulability
CTA: Computed tomographic angiography
CT: Computed tomography
APTT: Activated partial thromboplastin time
PT: Prothrombin time
FDP: Fibrin degradation products
MRI: Magnetic resonance imaging
DWI: Diffusion weighted imaging
RLS: Right-to-left cardiac shunt
CRP: C-reactive protein
CA: Carcinoma antigen
CEA: Carcinoembryonic antigen
DIC: Disseminated intravascular coagulation
EGFR: Epidermal growth factor receptor
LMWH: Low molecular weight heparin
TF: Tissue factor
NBTE: Non-bacterial thrombotic endocarditis
TTS: Three Territory Sign
TTI: Three Territory Infarcts
AF: Atrial fibrillation
NOACs: Novel oral anticoagulants
